# Assessing Hepatic Steatosis Following Weight Loss in Adolescents with Severe Obesity: A Randomized Controlled Trial

**DOI:** 10.3390/children12121652

**Published:** 2025-12-05

**Authors:** Ali Talib, Fien De Boom, Yvonne Roebroek, Givan Florian Paulus, Ger Koek, Simon Robben, Bjorn Winkens, Nicole Bouvy, Ernst van Heurn

**Affiliations:** 1 Department of Surgery, Maastricht University Medical Center, P.O. Box 616, 6200 MD Maastricht, The Netherlandssimon.robben@mumc.nl (S.R.); 2 NUTRIM School for Nutrition and Translational Research in Metabolism, Maastricht University, 6211 LK Maastricht, The Netherlands; bjorn.winkens@maastrichtuniversity.nl (B.W.); n.d.bouvy@lumc.nl (N.B.);; 3 Department of General Surgery, Maxima Medical Center, 5504 DB Veldhoven, The Netherlands; 4 Department of General Surgery, Spaarne Gasthuis, 2134 TM Hoofddorp, The Netherlands; 5 Department of Methodology and Statistics, CAPHRI Care and Public Health Research Institute, Maastricht University, 6200 MD Maastricht, The Netherlands; 6 Department of Surgery, Leiden University Medical Centers, 2333 ZA Leiden, The Netherlands

**Keywords:** severe obesity, bariatric surgery, MASLD, hepatic steatosis

## Abstract

Background/Objectives: To assess whether laparoscopic adjustable gastric banding (LAGB) and combined lifestyle intervention (CLI) reduce hepatic steatosis more effectively than CLI alone in adolescents with severe obesity. Methods: Adolescents aged 14–16 with a BMI ≥ 40 kg/m^2^ (or ≥35 kg/m^2^ with comorbidity) were randomized to receive LAGB + combined lifestyle intervention (CLI, *n* = 30) or CLI alone (*n* = 30). Hepatic fat was assessed at baseline and one year via ultrasound-based Hepatorenal Index (HRI), liver span, and ALAT levels. Results: Of 59 participants (mean age 15.7, 80% female, BMI 44.3 kg/m^2^), 58.9% had steatosis at baseline (HRI ≥ 1.40). After one year, BMI decreased by 5.6 kg/m^2^ in the LAGB group but remained stable in controls. Steatosis resolution (HRI < 1.05) occurred in 21.4% of LAGB versus 4.4% of CLI patients (*p* = 0.078). Liver span declined by 1.09 cm post-LAGB (95% CI −2.05 to −0.13) and correlated with HRI improvement. ALAT levels were unchanged. Conclusions: LAGB led to greater reductions in hepatic fat and size than lifestyle changes alone. Though steatosis resolution was not statistically significant, findings suggest bariatric surgery may be a promising strategy for mitigating early hepatic changes in severe adolescent obesity. What is already known: Severe obesity in adolescents is frequently accompanied by hepatic steatosis, which can progress to metabolic dysfunction–associated steatotic liver disease (MASLD). Bariatric procedures—such as laparoscopic adjustable gastric banding (LAGB)—are proven to induce substantial weight loss and improve obesity-related comorbidities in youth. What this study adds: This is the first randomized controlled trial to evaluate the effect of LAGB on hepatic steatosis specifically in adolescents. At one year, 21.4% of LAGB-treated patients no longer met the sensitive HRI cut-off for steatosis (<1.05) versus 4.4% of controls. Moderate weight loss after LAGB corresponded with significant improvements in the Hepatorenal Index and liver span, suggesting a reduction in hepatic fat content.

## 1. Introduction

The global surge in severe obesity among adolescents has led to a marked increase in comorbid conditions such as type 2 diabetes, hypertension and metabolic dysfunction–associated steatotic liver disease (MASLD), of which hepatic steatosis is a hallmark feature [1,2]. This trend underscores the pressing need for robust diagnostic modalities and effective interventions tailored to this vulnerable age group. Bariatric procedures—including laparoscopic adjustable gastric banding (LAGB)—have proven effective for achieving substantial weight loss and ameliorating obesity-related complications in adolescents [3,4].

Hepatic steatosis—defined by intrahepatic fat accumulation exceeding 5%—poses a significant concern in youth, given its potential progression to steatohepatitis and cirrhosis [5,6,7]. Yet, despite the clinical importance of fatty liver in the context of adolescent obesity, data on its prevalence and natural history following bariatric surgery remain sparse [8]. Notably, no randomized controlled trials have addressed how surgical weight loss impacts hepatic fat content in this population.

In this trial, we employ the noninvasive hepatorenal index (HRI) to quantify changes in hepatic steatosis among adolescents with severe obesity randomized to LAGB plus combined lifestyle intervention (CLI) versus CLI alone. While liver biopsy remains the definitive diagnostic standard, HRI offers a practical, bedside tool with well-validated cut-offs that optimize sensitivity for exclusion (<1.05) or specificity for inclusion (≥1.40) of significant steatosis [9,10,11].

By elucidating the trajectories of liver fat reduction post-surgery, our findings will inform both screening strategies and therapeutic decision-making for hepatic steatosis in adolescents with severe obesity—an at-risk group for whom long-term liver health is of paramount concern.

## 2. Methods

**Figure 1:**
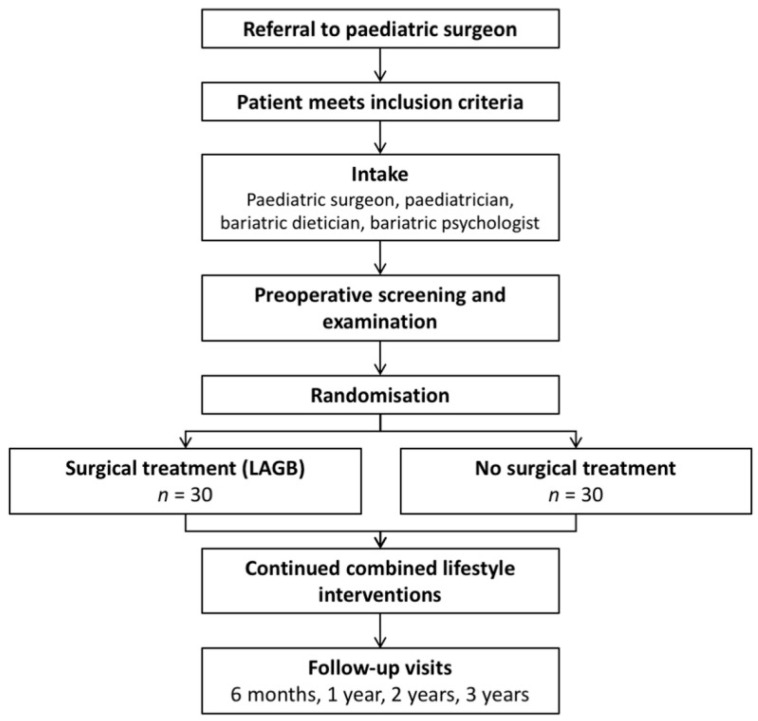
Flow Diagram of the Study.

This study was conducted as part of a randomized controlled trial for adolescents eligible for bariatric surgery (BASIC trial, NCT01172899) [3]. This study was registered in the ClinicalTrials.gov registry on 30 July 2010, with the identifier NCT01172899. The study population of the BASIC trial consists of adolescents with severe obesity who all have been treated extensively for their severe obesity by conservative methods for at least 12 months without effect. A flowchart detailing the BASIC trial study design is provided in Figure 1. 

### 2.1. Participants

Detailed information regarding the BASIC trial study design, inclusion and exclusion criteria, and randomization process was published previously [3]. The Medical Research Ethics Committee of the Maastricht University Medical Center approved the protocol for this study. In summary, inclusion criteria were age 14–16 years; sex- and age-adjusted BMI ≥ 40 kg/m^2^ (or ≥35 kg/m^2^ combined with the presence of obesity-associated comorbidity); and participation in combined lifestyle interventions during at least 12 months without adequate weight loss (defined as 5% total body weight loss). In order to maintain a homogenous study population with regard to pubertal status, girls were excluded if they were premenarchal and boys if their bone age was <15 years. All participants were subjected to standardized comprehensive baseline measurements and investigations in order to exclude (subclinical) conditions causing obesity. Participant inclusion started in December 2011 and continued until April 2019. Written informed consent was obtained from all participants and parents or guardians. 

### 2.2. Intervention and Control

Participants in the intervention group received a laparoscopic placement of an adjustable gastric band (LAP-BAND AP Adjustable Gastric Banding System with OMNIFORM Design; Allergan, Santa Barbara, CA, USA). The surgical procedures were performed by two surgeons: one leading bariatric surgeon and a second surgeon trained by the leading surgeon, both using the same surgical protocol (pars flaccida technique). Participants in both the intervention and control groups received continued CLI at their referring pediatric obesity clinic.

### 2.3. Measurements

All measurements within one patient were carried out during a single visit at baseline and after a one-year follow-up. Body height and weight were measured using a stadiometer and digital scale, respectively, with patients dressed in underwear. A tape measure was used for standardized measurement of body circumferences at neck level, abdominal level, and hip level. BMI was calculated as [body weight]/[body height × body height] in kg/m^2^, and BMI *z*-scores were calculated using Cole’s LMS method [12]. Daytime blood pressure was measured while the patient was resting, during a period of 60 to 90 min with intervals of 3 min between measurements, using the Mobil-O-Graph^®^ NG (I.E.M. GmbH, Stolberg, Germany). Prehypertension and hypertension were defined according to the fourth report from the National High Blood Pressure Education Program, and blood pressure *z*-scores were calculated according to the method described in that same report [13]. A fasting blood draw was performed to measure multiple lab parameters, including ALAT. ALAT was categorized as normal or elevated using the conventional stringent adult cut-off for the upper limit of normal of 40 U/L [14].

All ultrasounds to determine liver status, including HRI, were planned to be performed by a single pediatric radiologist with 30 years of experience in pediatric ultrasonography (SR). Ultrasonography was performed using the iU22 ultrasound system (Philips Healthcare, Eindhoven, The Netherlands) using a broadband C5-1 curved-array transducer. To determine the HRI, the probe was placed in a right subcostal or intercostal position, and three sagittal images were obtained of the parenchyma of the liver and kidney. A purely parenchymatous region of interest was selected for both organs in each image. The mean gray-scale intensity of the liver and renal parenchyma was established, and the average of three measurements was used to calculate the HRI using the following formula:

$$\mathrm{HRI}=\frac{\mathrm{echogenicity}\;\mathrm{of}\;\mathrm{the}\;\mathrm{liver}}{\mathrm{echogenicity}\;\mathrm{of}\;\mathrm{the}\;\mathrm{kidney}}$$

HRI was classified as low (<1.05), intermediate (1.05–1.39) and high (≥1.4), adopting the respective cut-offs suggested by Johnson et al. and Petzold et al. [9,10]. Liver length was measured in the midclavicular region. Hepatomegaly was defined as a midclavicular liver length ≥ 14.5 cm, which is the established upper limit of normal for the age group of 15–17 years [15].

### 2.4. Statistical Analysis

Numerical data are presented as mean ± standard deviation. Categorical data are presented as number (percentage). Demographic and clinical variables were compared between the intervention and control arms using an independent samples *t*-test for numerical variables and chi-square or Fisher’s exact test for categorical variables. All assumptions were checked using plots (histograms, qq-plots). IBM SPSS Statistics for Windows (version 28.0; Armonk, NY, USA) was used for the aforementioned statistical analyses. A two-sided *p*-value ≤ 0.05 was considered statistically significant.

## 3. Results

### 3.1. Baseline Study Population

A total of 60 adolescents were randomized in equal blocks to the intervention and control arms; one participant in the intervention group was subsequently excluded after being diagnosed with a prolactinoma. The remaining 59 patients (Table 1) had a mean age of 15.7 ± 1.0 years and were predominantly female (80%). Baseline obesity was severe, with a mean BMI of 44.3 ± 5.3 kg/m^2^ and a BMI z-score of 3.53 ± 0.29. Hepatorenal Index (HRI) measurements were obtained in 55 of 59 patients (93.2%). Due to unavailability, five exams were performed by a second experienced pediatric radiologist. The mean HRI was 1.56 ± 0.47. Using established cut-offs, 6 patients (10.9%) had low HRI (<1.05), 16 (29.1%) fell into the intermediate range (1.05–1.39), and 33 (60.0%) met the criterion for high HRI (≥1.40).

### 3.2. Weight Loss

At one year (Table 2), the LAGB arm achieved a mean BMI reduction of 5.24 kg/m^2^ (95% CI −6.57 to −3.91), whereas controls saw a slight increase of 0.37 kg/m^2^ (95% CI −1.21 to 1.96). This translated into a highly significant between-group difference of −5.61 kg/m^2^ (95% CI −7.61 to −3.61; *p* < 0.001). Nonetheless, the intervention cohort’s mean BMI remained 39.0 ± 6.9 kg/m^2^ at follow-up—still within or bordering the threshold for severe obesity.

### 3.3. Hepatorenal Index

At one-year follow-up, mean HRI in the LAGB arm was 1.45 ± 0.42 versus 1.62 ± 0.54 in controls, corresponding to an adjusted between-group difference of −0.23 (95% CI −0.55 to 0.09) (Table 2).

**Figure 2:**
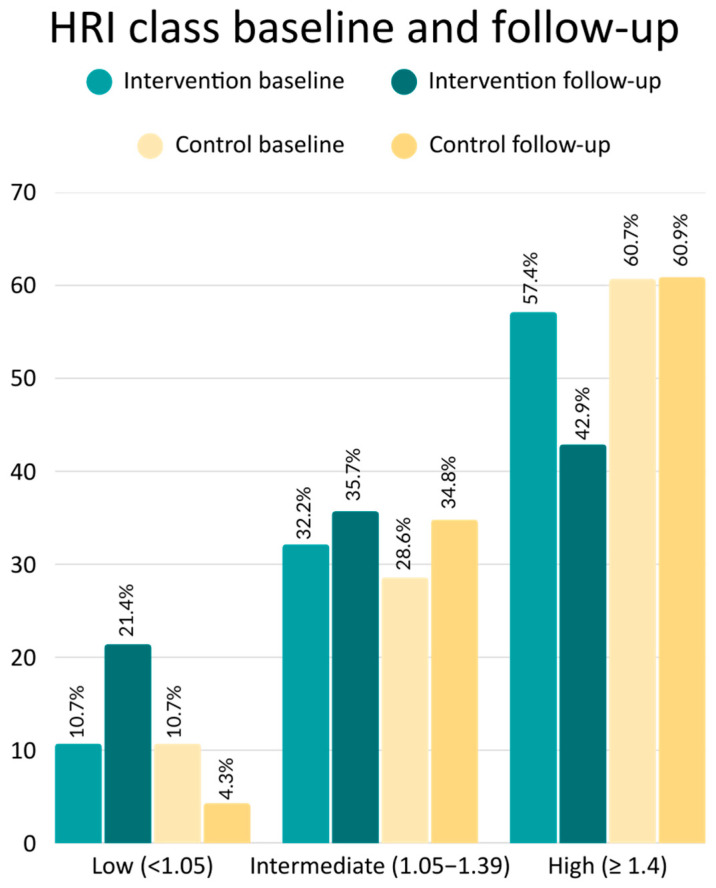
Percentage of patients with hepatic steatosis at different cut-off levels at baseline and follow-up. Differences were not statistically significant.

When categorized (Table 2 and Figure 2), 6 of 28 operated patients (21.4%) achieved HRI < 1.05—indicating steatosis exclusion—versus 1 of 23 controls (4.3%), an absolute difference of 17.1% (*p* = 0.078). Conversely, 12/28 (42.9%) in the intervention group and 14/23 (60.9%) of controls remained at HRI ≥ 1.40—a 14.4% difference (*p* = 0.200).

### 3.4. Other Surrogates for Hepatic Steatosis

Mean mid-clavicular liver length at one year was significantly smaller in the LAGB arm (13.46 ± 2.29 cm) than in controls (15.02 ± 2.53 cm; *p* = 0.024). However, the between-group difference in change from baseline (−0.93 cm; 95% CI −2.38 to 0.53) did not reach significance (*p* = 0.206). At follow-up, HRI and liver length were modestly correlated (Pearson r = 0.302, *p* = 0.031). In a post hoc linear regression adjusting for sex and BMI, HRI remained an independent predictor of liver length (B = 2.035 cm per HRI unit, SE = 0.791, *p* = 0.013).

By contrast, plasma ALAT levels at one year were virtually identical between groups (*p* = 0.991) and showed no relationship with HRI (r = 0.01, *p* = 0.946). Likewise, in an adjusted regression model, HRI bore no significant association with ALAT (B = −1.883 U/L, SE = 6.390, *p* = 0.770).

## 4. Discussion

This randomized controlled trial is the first to evaluate changes in hepatic steatosis among adolescents with severe obesity following bariatric surgery—a high-risk cohort that has received little attention in prior research. At baseline, we observed an alarmingly high prevalence of hepatic steatosis in both study arms. By the end of follow-up, a greater proportion of patients in the surgery group demonstrated resolution of steatosis (21.4%) compared with those in the control group (4.3%), although it was not statistically significant (*p* = 0.078).

### 4.1. Validity of HRI for Hepatic Steatosis

Histopathology after liver biopsy remains the definitive method to diagnose and quantify hepatic steatosis (≥5% liver fat), but its invasiveness and cost limit its use. MRI and ^1^H-MRS offer non-invasive alternatives with excellent accuracy, yet they too can be expensive and time-consuming [16].

**Figure 3:**
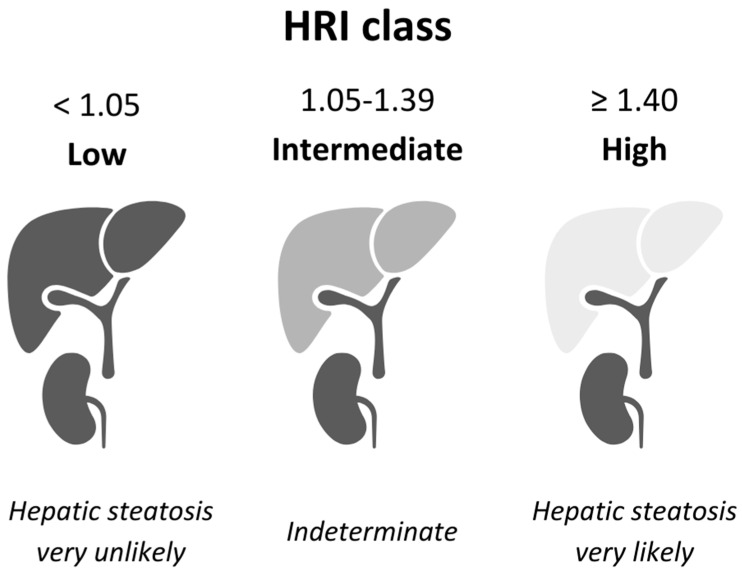
Visual depiction of the diagnostic significance within each HRI category, based on multiple validation studies including Johnson and Petzold [9,10].

The Hepatorenal Index (HRI) on ultrasound is a well-validated, widely studied surrogate for hepatic steatosis [9,10,17,18,19,20,21,22]. In our protocol we define:

- HRI < 1.05 to rule out steatosis (high sensitivity; Petzold et al. report 85.4% sensitivity);
- HRI ≥ 1.40 to confirm steatosis (high specificity; Johnson et al. report 99% specificity, albeit only 4% sensitivity, yielding a 95% positive predictive value);
- HRI 1.05–1.39 as indeterminate (see Figure 3).

These cut-offs are drawn from two recent biopsy-based studies—Johnson et al. in a diverse adult cohort [9] and Petzold et al. [10] in a high-risk adolescent population—and performed consistently well at the extremes of the HRI spectrum [23,24,25,26].

### 4.2. Baseline Prevalence of Hepatic Steatosis

In our cohort, applying an HRI threshold of ≥1.4 yielded a hepatic steatosis prevalence of 58.9%. While high, this figure is broadly in line with pediatric obesity data: Anderson et al. found a pooled steatosis prevalence of 34.2% (95% CI 27.8–41.2%), with individual studies ranging from 5.2% to 83.1% depending on age and BMI strata [8]. The particularly severe obesity in our sample (mean BMI 44.3 ± 5.3 kg/m^2^) likely underlies the elevated rate. Indeed, Xanthakos et al. reported a 59% prevalence of MASLD in a comparable group of predominantly White, severely obese adolescent females undergoing bariatric surgery [7], essentially mirroring our finding. For context, adult autopsy series document steatosis in approximately 65% of individuals with BMI 30.0–39.9 kg/m^2^ and 85% of those with BMI ≥ 40 kg/m^2^ [27].

### 4.3. Prevalence of Hepatic Steatosis on Follow-Up

The follow-up HRI decreased by 0.14 in the intervention group but increased by 0.09 in the control group, though this difference was not statistically significant (*p* = 0.858). When we applied the sensitive cut-off of <1.05 at follow-up, steatosis was absent in 21.4% of the intervention arm versus 4.3% of controls (*p* = 0.078). Conversely, using the specific threshold of ≥1.40, hepatic steatosis persisted in 42.9% of operated patients compared with 60.9% of controls (*p* = 0.200). Although these *p*-values exceed 0.05—likely reflecting both our modest sample size and only moderate weight loss—mean BMI at follow-up remained 39.0 kg/m^2^ in the intervention group (just below the severe obesity cut-off) versus 45.4 kg/m^2^ in controls, which may explain the continued high prevalence of steatosis even after surgery.

### 4.4. Midclavicular Liver Length and ALAT

We included midclavicular liver length and serum alanine aminotransferase (ALAT) as secondary, noninvasive surrogates for MASLD, given their established positive correlations with hepatic fat content [28,29,30,31]. Although hepatomegaly and elevated transaminases can arise from other liver or cardiac diseases, none of our participants had viral hepatitis, focal lesions, or heart failure, making fatty infiltration the most likely driver of any observed changes.

Within the intervention arm, mean midclavicular liver length fell by 1.09 cm (95% CI −2.05 to −0.13), whereas the control arm showed a nonsignificant 0.16 cm decrease (95% CI −1.32 to 1.00). A subsequent linear regression confirmed that HRI remained independently associated with liver length after adjusting for BMI and sex, supporting the notion that liver size reduction post-LAGB reflects genuine decreases in steatosis.

We also measured ALAT—an indicator of hepatocellular injury that, while not diagnostic, often parallels steatosis severity in adults [31]. Using the conventional adult upper limit of normal (40 U/L) [14], we found no significant difference in ALAT between groups at one year. We suspect this reflects the predominance of simple steatosis rather than steatohepatitis in adolescents—a pattern supported by pediatric autopsy series demonstrating minimal inflammatory change in youth with fatty liver [32].

## 5. Study Limitations

Our study does have several notable limitations. First, the relatively small sample size inherent to this exploratory design may have limited our ability to detect statistically significant differences for certain outcomes. Second, although almost all ultrasounds were performed by a single highly experienced pediatric radiologist, a few were interpreted by a different radiologist when the primary operator was unavailable; while published data suggest low inter-observer variability for HRI and mid-clavicular liver length measurements [17,33], subtle bias cannot be entirely excluded. Third, as previously noted, the sensitivity of an HRI value below 1.05 is adequate but suboptimal (inferior to 85.4%), which may result in the underestimation of the prevalence of hepatic steatosis [10]. Fourth, in a minority of cases, HRI and mid-clavicular liver length were not documented in the ultrasound reports despite protocol, possibly reflecting technical challenges—such as poor acoustic windows in individuals with very high BMI—but we could not ascertain the exact reasons, especially for scans read by the secondary radiologist. Future prospective studies should record the feasibility of these measurements and the rationale for any omissions. Finally, our one-year follow-up may have been too brief to capture the full trajectory of steatosis resolution after bariatric surgery, underscoring the need for longer-term investigations.

Key messages

- Hepatic steatosis affects the majority of adolescents with severe obesity: in our cohort, 58.9% met the highly specific HRI threshold of ≥1.40 for steatosis.
- One year after laparoscopic adjustable gastric banding (LAGB), moderate weight loss corresponded with reductions in liver fat: using the sensitive exclusion cut-off (HRI < 1.05), steatosis resolved in 21.4% of operated patients versus 4.4% of controls (baseline prevalence 10.7% in both; *p* = 0.078).
- Approximately one-third of adolescents with severe obesity fall into an indeterminate HRI range (1.05–1.39). In practice, these patients warrant further evaluation—such as assessment of hepatomegaly or transaminase levels—to guide management, and confirmation of steatosis might support consideration of bariatric surgery even in class II obesity.

## Figures and Tables

**Table 1:** Baseline characteristics.

Variable	Overall (*n* = 59)	Intervention Arm (*n* = 29)	Control Arm (*n* = 30)
Patient Age (Years)	15.74 (±1.01)	15.90 (±1.01)	15.58 (±1.01)
Sex: female	47 (80%)	23 (79%)	24 (80%)
Length (Meters)	1.70 (±0.09)	1.71 (±0.09)	1.70 (±0.08) (*n* = 30)
Weight (Kilograms)	128.76 (±19.82)	128.96 (±18.51)	128.57 (±21.31) (*n* = 30)
BMI (kg/m^2^)	44.27 (±5.25)	44.25 (±4.97)	44.29 (±5.59) (*n* = 30)
BMI Deviation in Age and Sex Group	3.53 (±0.29)	3.53 (±0.27)	3.54 (±0.31)
Hepatorenal Index (HRI)	1.56 (±0.47)	1.57 (±0.54) (*n* = 27)	1.55 (±0.40) (*n* = 28)
Hepatorenal Index High (≥1.4) (*n* = 56)	33 (58.93%)	16 (57.14%)	17 (60.71%)
Hepatorenal Index Low (<1.05) (*n* = 56)	6 (10.71%)	3 (10.71%)	3 (10.71%)
Liver Midclavicular Length (cm)	14.72 (±2.34)	14.54 (±2.69) (*n* = 28)	14.90 (±1.95) (*n* = 28)
ALAT (U/L) (*n* = 59)	30.92 (±15.49)	29.14 (±14.66) (*n* = 29)	32.63 (±16.30) (*n* = 30)

Continuous variable values are reported as mean ± SD and categorical ones as number of patients (%).

**Table 2:** Overview comparing variables within and between intervention and control arms.

		Baseline (Mean ± SD)	Follow-Up (Mean ± SD)	Change Within Group (Mean ± 95% CI)	Difference Between Groups (Mean ± 95% CI)	Intervention Effect (*p*)
Weight (kg)	Intervention (*n* = 29)	128.96 (±18.51)	114.67 (±20.31)	−14.29 (−18.15–−10.42)	−16.76 (−22.72–−10.81)	**<0.001**
	Control (*n* = 23)	131.52 (±21.74)	134.00 (±26.48)	2.48 (−2.35–7.31)		Reference
BMI (kg/m^2^)	Intervention (*n* = 29)	44.25 (±4.97)	39.01 (±6.87)	−5.24 (−6.57–−3.91)	−5.61 (−7.61–−3.61)	**<0.001**
	Control (*n* = 23)	45.02 (±5.72)	45.39 (±6.68)	0.37 (−1.21–1.96)		Reference
BMI Z-score	Intervention (*n* = 29)	3.53 (±0.27)	3.09 (±0.55)	−0.44 (−0.58–−0.30)	−0.44 (−0.60–−0.27)	**<0.001**
	Control (*n* = 23)	3.58 (±0.30)	3.58 (±0.36)	0.003 (−0.09–0.10)		Reference
Waist Circumference (cm)	Intervention (*n* = 26)	125.67 (±12.24)	114.85 (±15.68)	−10.83 (−14.96–−6.69)	−9.02 (−15.90–−2.15)	**0.011**
	Control (*n* = 21)	130.28 (±14.55)	128.48 (±17.70)	1.80 (−7.80–4.19)		Reference
Hepatorenal Index (HRI)	Intervention (*n* = 26)	1.59 (±0.55)	1.45 (±0.42)	−0.14 (−0.38–0.09)	−0.23 (−0.55–0.09)	0.858
	Control (*n* = 21)	1.53 (±0.41)	1.62 (±0.54)	0.087 (−0.15–0.33)		Reference
Hepatorenal Index High (≥1.4)	Intervention	16 (57.14%) (*n* = 28)	12 (42.86%) (*n* = 28)	−14.28%	−14.45%	0.200 *
	Control	17 (60.71%) (*n* = 28)	14 (60.87%) (*n* = 23)	+0.17%	-	
Hepatorenal Index Low (<1.05)	Intervention	3 (10.71%) (*n* = 28)	6 (21.43%) (*n* = 28)	+10.72%	17.08%	0.078 *
	Control	3 (10.71%) (*n* = 28)	1 (4.35%) (*n* = 23)	−6.36%	-	
Liver Midclavicular Length (cm)	Intervention (*n* = 28)	14.54 (±2.69)	13.45 (±2.34)	−1.09 (−2.05–−0.13)	−0.93 (−2.38–0.53)	0.206
	Control (*n* = 21)	14.84 (±2.01)	14.68 (±2.37)	−0.16 (−1.32–1.00)		Reference
ALAT (U/L)	Intervention (*n* = 26)	29.50 (±15.32, *n* = 26)	26.23 (±18.81, *n* = 26)	−3.27 (−7.07–13.60)	−0.07 (−12.89–12.75)	0.991
	Control (*n* = 25)	34.72 (±16.62)	31.52 (±16.45)	−3.20 (−4.81–11.21)		Reference

Intervention effects were determined via independent samples T-tests comparing the mean variable change between the intervention and control arms; bold 95% confidence intervals indicate significance. * Pearson chi-square analysis.

## Data Availability

The raw data supporting the conclusions of this article will be made available by the authors on request.
